# Seroepidemiology of Human Enterovirus 71, Singapore

**DOI:** 10.3201/eid0809.10.3201/eid0809.010397

**Published:** 2002-09

**Authors:** Eng-Eong Ooi, Meng-Chee Phoon, Baharudin Ishak, Soh-Ha Chan

**Affiliations:** * National Environment Agency, Singapore; † National University of Singapore, Singapore

**Keywords:** HEV71, serologic survey, transmission, preschool-aged children

## Abstract

Human enterovirus 71 has caused outbreaks in many parts of the world, especially Southeast Asia, with some fatal cases. The epidemiology of this viral infection is not well understood. We conducted a serologic survey in Singapore children, and the results indicate that infection occurs largely in preschool settings.

Human enterovirus 71 (HEV71) is an emerging concern in many parts of the world. It has caused several large outbreaks, occasionally associated with many deaths in children (1–3). In September and October 2000, a large nationwide outbreak of hand, foot, and mouth disease (HFMD) caused by HEV71 occurred in Singapore (4). Most of the cases were in children <6 years of age; four cases were fatal.

The epidemiology of HEV71 infection in Singapore and most parts of the world has not been well studied. A few reports suggest that HEV71 infection is common and mostly subclinical (5,6). Differences in the DNA sequences of the HEV71 isolates do not appear to play an important role in clinical outcome (7,8). Ho and colleagues (3) cited preliminary evidence indicating that more than half the adult population in Taiwan had been exposed to HEV71 before the 1998 epidemic.

The settings where most HEV71 transmission occurs are, however, uncertain. During the 2000 outbreak of HEV71-associated HFMD in Singapore, a decision was made to close all preschool centers nationwide in an effort to break the chain of transmission of this virus (4). The effectiveness of this control measure is unclear. HFMD was made a legally notifiable disease on October 1, 2000, which coincided with both the middle of the HEV71-associated HFMD outbreak and the closing of the preschool centers in Singapore (4). Although the closure of preschool centers was thus temporally associated with a decline in reported HFMD cases, no comparable data were available before and after the implementation of the preschool closure to allow assessment of the impact of this measure.

To devise appropriate preventive measures, the transmission of this virus in the natural setting needs to be determined. A serologic survey would be useful for this purpose. Such a study in children had not been conducted in this region. We report the findings of an HEV71 serologic survey in Singapore children.

## The Study

We surveyed 856 children <12 years of age. Serum samples were collected, with informed parental consent, during the 18-month period of July 1996 to December 1997 at a pediatric clinic at the National University Hospital, which serves the entire country. All children who were born at the hospital or who were brought for routine visits and vaccinations during this period were included. The children had no sign of disease at the time of sample collection.

The serum samples, which had been used in a previous study of dengue (9), were divided and stored at –20°C and inactivated at 56°C for 30 min before use. Neutralizing antibody against HEV71 (EV71/7423/MS/87), an isolate that had been characterized previously (10), was detected by using a neutralization test by microtechnique as previously described, with modifications (11). Sample dilutions of 1:8 to 1:2,048 were assayed. Twenty-five microliters of 100 tissue culture infective dose (TCID_50_) virus was mixed with 25 μL of the appropriate serum dilution and incubated for 2 h at 37°C in a CO_2_ incubator, followed by the addition of 100 μL of rhabdomyosarcoma cell suspension at a concentration of 2 x 10^4^ cells/0.1 mL. Each dilution was tested in duplicate. Readings were taken visually with an inverted microscope after 6 days of incubation at 37°C in 5% CO_2_. The antibody titer for the sample is the highest dilution that prevents the development of cytopathic effects in both wells. An antibody titer of >8 was considered positive. The geometric mean titer (GMT) and 95% confidence intervals (95% CI) were also calculated. Statistical analysis was done by Student t test.

**Figure 1:**
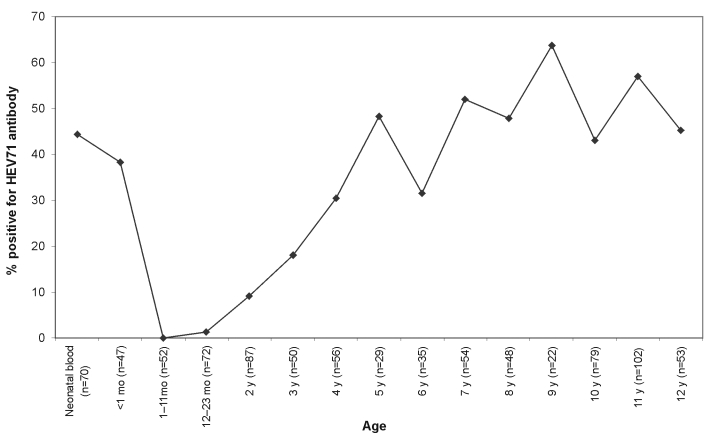
Seroprevalence rate of anti-Human Enterovirus 71 (HEV71) antibodies.

The results showed that 44.0% of mothers had antibodies to HEV71 (deduced from antibody prevalence in cord blood), which waned rapidly so that after 1 month, none of the children tested had maternal antibodies to HEV71 (Figure 1). Only 1 (0.8%) of the 124 samples from children ages 1–23 months had anti-HEV71 antibodies. From 2 to 5 years, the seropositive rate increased at an average of 12% per year. In samples from children >5 years old, the age-specific seroprevalence reached a steady state at approximately 50%.

**Figure 2:**
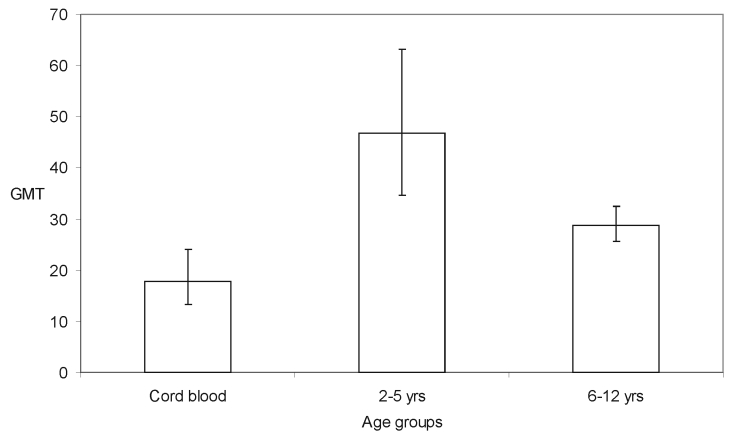
Geometric mean titer (GMT) of anti-Human Enterovirus 71 (HEV71) antibody with 95% confidence intervals in children of different age groups.

Figure 2 shows the age-specific GMT in seropositive samples by age group. Formal schooling in Singapore begins at 6 years of age. From 2 to 5 years, however, most children receive preschool education in child-care centers or kindergartens. The GMT of the anti-HEV71 antibodies was higher in preschool children (GMT 46.8; 95% CI 34.7 to 63.1) than in those of formal school age (6–12 years old) (GMT 28.8; 95% CI 25.7 to 32.4). The difference in these two means was statistically significant (t test = 3.07; df = 240; p=0.002). Furthermore, the GMT titer of maternal antibody in neonatal blood (GMT 17.8; 95% CI 13.2 to 24) was also significantly lower than in the preschool children (t test = 4.02; df = 78; p=0.0001).

## Conclusions

This report is the first detailing the seroepidemiology of HEV71 in Singapore children. The results indicate that most infection occurs in preschool-aged children. The concentration of this susceptible group of the population in classrooms, along with the sharing of toys and other teaching tools, are factors contributing to HEV71 transmission.

The differences in GMT in the different age groups indicate that infection outside these preschool years is uncommon. This finding is supported by the following observations: 1) the small proportion of children <2 years old who were seropositive; 2) the proportion of seropositive children reached a steady state after 5 years of age; and 3) the GMT of anti-HEV71 antibody declined with age. If substantial levels of transmission occurred at home, GMT would be expected to increase with age, as reexposure to HEV71 would boost the antibody levels in both children and mothers. Transmission at home would also result in high maternal antibody titers that last for at least 6 months, as observed in our previous study of dengue (9). Furthermore, a fair proportion of children <2 years old would also be expected to be seropositive. Instead, the results of this study indicate that HEV71 is transmitted in preschool centers, where older children and adults are less likely to spend substantial amount of time. The control of HEV71 infection should thus be specifically focused at such places. The cost-effectiveness of implementing preventive measures such as strict observation of handwashing in a preschool setting in Singapore should also be evaluated.

For this study, we used serum samples collected during 1996–1997. In 1997, an outbreak of HEV71 in children occurred in Malaysia that resulted in several deaths (1,2). In Singapore at that time, HFMD was monitored on the basis of volunteer reporting from the operators of preschool centers. This surveillance allowed 12 localized clusters of HFMD to be identified (12). Virus isolation studies were also carried out on samples from the children who attended these centers. Coxsackie virus A16 was isolated in three of these clusters, and HEV71 was isolated in one. No viruses were isolated in the other clusters. No HEV71-related deaths were recorded during this period. These observations indicate that HEV71 was present during or before the time of sample collection, although no nationwide outbreak was observed during that time.

This study is confined in scope to Singapore children. To support the hypothesis that HEV71 transmission occurs largely in the preschool setting, a serologic study of preschool teachers and directors would be very useful. Furthermore, as the mode of transmission of enteroviruses is essentially the same, by the fecal-oral route, the serologic profile for the other enteroviruses may be the same as that observed in this study. No such data are currently available.

In conclusion, HEV71 infection is common in Singapore children and is acquired largely in the preschool years. Transmission of this virus is lower in the other age groups. This finding suggests that HEV71 transmission occurs mainly in places where preschool children congregate, and public health measures to control the spread of this virus should focus on these places.
